# REACH Specific Environmental Release Categories for Plant Protection Product Applications

**DOI:** 10.1002/ieam.4251

**Published:** 2020-03-25

**Authors:** Christopher Dobe, Sébastien Bonifay, Joachim D Krass, Claire McMillan, Adrian Terry, Matthias Wormuth

**Affiliations:** ^1^ Syngenta Crop Protection AG Basel Switzerland; ^2^ Corteva Agriscience, Production Agriscience Belgium BVBA Brussels Belgium; ^3^ BASF SE Ludwigshafen Germany; ^4^ Cambridge Environmental Assessments Cambridge United Kingdom

**Keywords:** REACH, Exposure modeling, Environmental release category, SpERC, Pesticide

## Abstract

The European Registration, Evaluation, Authorisation and Restriction of Chemicals (REACH) regulation requires that quantitative environmental risk assessment is carried out for hazardous substances used as coformulants in plant protection products (PPPs), if registered above 10 t/y. The European Crop Protection Association (ECPA) has developed generic exposure scenarios and specific environmental release categories (SpERCs) to support these risk assessments. The SpERCs offer refinements to the default release factors defined in environmental release categories (ERCs) and are intended to be used with nested multimedia mass balance models as part of the assessment of regional predicted environmental concentrations. Based on the application method of PPPs, 2 scenarios were defined for which SpERCs were developed: 1) spraying of PPPs and 2) direct application of granular products or treated seeds to soil. The SpERC for spray applications includes release factors to air and soil that depend on the vapor pressure of the coformulant. Calculations are presented to support the subSpERCs describing the transition from nonvolatile to volatile behavior. The most recent version of the spray application SpERC defines a release factor for surface water and more conservative release factors to soil compared with previous versions. Use of the ECPA SpERCs allows the coformulant emissions from PPPs to be fully accounted for in the regional‐scale environmental risk assessment for a given substance, along with all other sources of emissions. Qualitative and quantitative justification for the ECPA‐derived SpERCs is presented and serves as the background documentation to the online European Chemicals Agency (ECHA) SpERC factsheets. The approach developed here whereby regional‐scale SpERCs are used in combination with a customized local‐scale exposure model is potentially applicable for other sectors that are required to conduct exposure assessments outside the scope of the standard environmental REACH models. *Integr Environ Assess Manag* 2020;16:472–480. © 2020 Syngenta Crop Protection AG. *Integrated Environmental Assessment and Management* published by Wiley Periodicals, Inc. on behalf of Society of Environmental Toxicology & Chemistry (SETAC)

## KEY POINTS

- Specific environmental release categories (SpERCs) have been developed to support the environmental risk assessment of coformulants used in plant protection products under the European Registration, Evaluation, Authorisation and Restriction of Chemicals (REACH) regulation.
- The SpERCs were developed for 2 scenarios: spraying of plant protection products and direct application of granular products or treated seeds to soil.
- Use of these SpERCs allows the coformulant emissions from plant protection products to be fully accounted for in the regional‐scale exposure assessment, along with all other sources of emissions.

## INTRODUCTION

European Regulation (EC) Nr 1907/2006 (Registration, Evaluation, Authorisation and Restriction of Chemicals [REACH]) (EC 2006) requires registrants in the European Economic Area to submit a chemical safety report for substances manufactured or imported at quantities greater than 10 t/y. For substances classified as hazardous, an exposure assessment and risk characterization is required for all identified uses, including use as coformulants in plant protection products (PPPs). Coformulants (assumed here for brevity to be substances, but are frequently also mixtures) are defined as all the intentionally added components of the formulation, other than the active substance, for example, solvents, surfactants, colorants, fillers, and antifoams. As such, coformulants are often commodity chemicals with a multitude of nonPPP uses, whereas the use in PPPs is often a niche application in terms of the overall tonnage manufactured. For coformulants that are hazardous and demonstrate environmental effects, the corresponding chemical safety assessment must contain an environmental exposure assessment that covers PPP uses and demonstrate that those uses are safe. The European Crop Protection Association (ECPA) has developed generic exposure scenarios for substances used as coformulants in PPPs, which enable manufacturers and importers to perform the required assessments in an efficient, robust, and standardized manner (Dobe et al. 2017).

The environmental risk assessments performed for REACH typically utilize a local‐scale model and nested multimedia mass balance models for the regional and continental scales (ECB 2003; RIVM 2004; Vermeire et al. 2005; ECHA 2016a), referred to generically here as EU technical guidance document (EU TGD) models, for which release factor estimation is a critical step for defining the mass released to each compartment. The models are relatively complex, and various interdependencies exist between the input parameters (Berding et al. 1999; Matthies et al. 2004).

Use descriptors (environmental release categories [ERCs]) have been developed by the European Chemicals Agency (ECHA) for grouping activities from the environmental perspective (ECHA 2015). Each category has been assigned default worst‐case release factors for use in the absence of more specific information (ECHA 2016a). The relevant ERC for describing the use of substances as coformulants in PPPs is ERC 8d: “Widespread use of non‐reactive processing aid (no inclusion into or onto article, outdoor).” The default release factors for ERC 8d are given in Table 1.

**Table 1:** Release factors for ERC 8d and ECPA SpERCs Version 4

Vapor pressure (Pa)	F_air_ (−)	F_soil_ (−)	F_surface water_ (−)
Release factors for ERC 8d: Widespread use of nonreactive processing aid			
N/A	1	1	0.2
Release factors for spray applications (SpERC 8d.2.4)			
≥0.01	1	0	0.002
0.001 to <0.01	0.5	1	0.002
0.0001 to <0.001	0.2	1	0.002
0.00001 to <0.0001	0.1	1	0.002
<0.00001	0.01	1	0.002
Release factors for application as granules or treated seeds (SpERC 8d.1.4).			
≥0.01	0	1	0
<0.01	0	1	0

ECPA = European Crop Protection Association; ERC = environmental release category; N/A = not applicable; Pa = Pascal; SpERC = specific environmental release category.

In response to the ERC release factor conservatism, several industry sector organizations have published specific environmental release categories (SpERCs), taking into account the operational conditions and risk management measures relevant for use of substances in those sectors (Sättler et al. 2012; Verdonck et al. 2014; Tolls et al. 2015; Reihlen et al. 2016; Ahrens et al. 2017). Similarly, SpERCs were developed by ECPA for the environmental exposure assessment of substances used as coformulants in PPPs. The SpERCs and associated release factors were developed for use solely with REACH nested multimedia mass balance models, as part of the regional scale and humans exposed via environment assessments, whereby emissions from all uses of a given substance are considered. Use of the ECPA SpERCs permits more realistic assessment of the environmental contributions from coformulant uses, and for these to be accounted for in the overall environmental risk assessment. The manufacture of PPPs is outside the scope of the ECPA SpERCs because formulation was considered to be a generic industrial activity not specific to the crop protection sector and already adequately covered by default ERCs or sector‐specific SpERCs, for example, European Solvents Industry Group SpERC 2.2.v2 Formulation and (re)packing of substances and mixtures (industrial) (ESIG 2018). It should be noted that the release factors defined in the ECPA SpERCs are not intended for assessments of local‐scale predicted environmental concentrations (PECs). The EU TGD‐based models as currently implemented do not consider direct emissions to air and agricultural soil for widespread uses at the local scale (ECB 2003; ECHA 2016a). As a consequence, ECPA has also developed a standalone model to address this gap, the Local Environment Tool (LET) (ECPA 2018), which should be used in conjunction with the EU TGD models. The LET uses the same basis for release factors implemented in the ECPA SpERCs in the calculation of PEC_local_. The approach developed here whereby regional‐scale SpERCs are used in combination with a customized local‐scale exposure model is potentially applicable for other sectors required to conduct environmental exposure assessments outside the local‐scale scope of the EU TGD models.

The present paper sets out the qualitative and quantitative justification for the ECPA‐developed SpERCs, including the most recent updated values (version 4), and aims to serve as the more detailed background documentation required for the summary SpERC factsheets hosted by ECHA in the Use Maps Library (ECHA 2016b, 2019b; Reihlen et al. 2016; Ahrens et al. 2017).

## METHOD

Specific environmental release categories can be justified in different ways (Ahrens et al. 2017). The approach taken here to derive the release factors used in the ECPA SpERCs was based around qualitative worst‐case arguments, combined with emission factors with previous regulatory acceptance, and where possible further supported by available data or calculations to demonstrate plausibility.

Four generic exposure scenarios were defined for the professional or consumer (amateur) use of substances as coformulants in PPPs (Dobe et al. 2017). However, based on application method, only 2 scenarios need to be differentiated in the environmental risk assessment: 1) spraying of diluted PPPs and 2) the direct application of granular products or treated seeds to soil. The assumption is that the physical state of the applied product (i.e., solid or liquid) is a key determinant to the extent to which the environmental compartments are exposed as a result of PPP uses. This should not be confused with the physical state of the substance. In the absence of any information on technical function or stability, a solid substance must be assumed to be potentially applied as both a spray and a granule. A liquid substance, on the other hand, can be assumed to have only a limited concentration in a potential granule or powder if the solid physical characteristics of the formulation are to be maintained, and in practice may never find such a use, for example, a solvent in a powder formulation.

Existing environmental exposure assessment models developed for active substances of PPPs provide a ready source of information on the exposure determinants that are potentially relevant (RIVM 1995, 2002; FOCUS 2001). The PPP application rate (kg/ha) is clearly the most important exposure determinant, being directly related to the mass emitted. Both spray drift (unintended, direct emission to surface water) and crop interception (the fraction of the applied PPP that is prevented from reaching the soil by the crop) are dependent on crop type, for example, vegetables, cereals, orchards. Vapor pressure is positively correlated with partitioning to air from both water and soil, although better correlations for volatilization have been proposed using Henry's law or alternative coefficients (Cousins et al. 1999). However, only vapor pressure is a standard data requirement for reporting under REACH, and it was used for this reason as an adequate descriptor for banding of volatility into subSpERCs.

The definition of a volatile substance is inherently relative to the timescale under consideration. For worker exposure, 10 Pascal (Pa) is often used as a trigger value, where the concern is the potential for significant atmospheric concentrations over a short duration (minutes to hours) (Fransman et al. 2011). In contrast, the environmental compartments within nested multimedia fate models are assumed to be homogeneous and well mixed. Emission is assumed to be constant over a period of time, and once emitted, chemicals are assumed to be immediately distributed and homogeneously mixed throughout the compartment. Temporal variation in use rates is accounted for in the EU TGDs by use of an assessment factor of 4 at the local scale. Considering the longer timescales involved in environmental assessments (hours to days), the vapor pressure at which a substance should be considered volatile is lower than for worker assessments, thus a cutoff of 0.01 Pa is commonly used (ECHA 2016a) and has been adopted here. Substances with vapor pressures above this can be assumed to fully and rapidly partition to air. It should be noted that the exposure durations used in the ecotoxicology studies upon which predicted no effect concentrations (PNECs) are based are at least between 48 and 96 h or longer, depending on whether long‐term studies are available.

The pesticides field application model in the Uniform System for the Evaluation of Substances (USES) 4.0 (RIVM 2002, Table A‐2, p 211 and Table D‐3, p 318) implemented vapor pressure–dependent release factors to air (F_air_). This USES module was an extension to the standard EU TGD, and was used for environmental assessments by the Dutch competent authority under previous national legislation for PPP registrations. The F_air_ values in USES 4.0 were derived from the averaged 24‐h emission strength, based on a 1 kg/ha application of a substance, assuming 90% of the emission occurred in the first day. Evaporation of volatile components, and coevaporation of less volatile components, from the very high surface area of a spray mist is rapid (Delmaar and Bremmer 2009). Because the release factor to air should describe the initial dynamic processes during spraying and shortly thereafter (RIVM 1995; FOCUS 2008), basing this value on a longer 24‐h period is thus extremely conservative. The derivation of the values in USES 4.0 appears to be based on the data reported by the Netherlands National Institute for Public Health and the Environmental (RIVM 1995).

A relation derived by van Wesenbeeck et al. (2008) from a data set of 82 active substances and coformulants was used to confirm the plausibility of the vapor‐pressure bands for the transition from nonvolatile to volatile behavior, the corresponding F_air_ values, and the 0.01 Pa cutoff for volatile substances:

(1) Ln(ER)=12.2+0.933×Ln(VP),

where ER is evaporation rate (µg/m^2^/h) and VP is vapor pressure (Pa). The pooled data set spanned a vapor pressure range of 0.0001 to >30 000 Pa, and was based on evaporation of pure substances and mixtures from plant, soil, and laboratory studies at ambient relative humidity (30%–50%). The vapor pressure range is particularly relevant for coformulants, whose vapor pressure ranges are often higher than active substances.

A data set was constructed to investigate the real‐world vapor pressure distribution of coformulants from 3 sources: 42 predominantly hazardous coformulants identified in an unpublished survey as being on the Irish market; a 180‐coformulant subset of the coformulants reported as being used in formulations approved for the German market; and an individual company data set of 96 substances. From this list, vapor pressure values were assembled from the ECHA REACH registration dissemination pages. Substances with no data, implausible values, or polymers were discarded, and based on chemistry 18 inorganic substances were assigned to the lowest vapor pressure band (e.g., TiO_2_), giving a final data set of 166 values.

Forum for the Co‐ordination of Pesticide Fate Models and their USe (FOCUS) ditch and stream scenarios, which are most relevant for regional‐scale exposure, assume that each 1‐ha agricultural field is adjacent to a 1‐m wide, 100‐m long water body (FOCUS 2001). These scenarios were combined with standard spray drift data to derive the ECPA SpERC release factor for surface water.

The EU TGD explicitly models runoff from soil to surface water at the regional and continental scale. Because emission to soil must occur first, and runoff from treated areas is dictated by a heavy rain event and is thus temporally separated from the initial spray emission, runoff should not be considered to contribute to the F_surface water_ release factors. Only direct emission that may result from spray drift is considered to be relevant to the ECPA SpERC release factor describing PPP‐related emission to surface water.

The realistic worst‐case spray drift, expressed as a percentage of the application rate, was assumed to be 15.7%, compared with the 2.8% expected for the majority of arable crops (FOCUS 2003). This realistic worst case corresponds to the 90th percentile spray drift values for citrus, olives, and late applications to pome and/or stone fruit (FOCUS 2001, 2003), and represents orchards where high spray drift may be expected. The remaining crops with higher drift values were not selected for use in the ECPA SpERC as being a realistic worst case at the regional scale (200 × 200 km): Early applications to stone and/or pome fruit (29.2%) are considered rare, hops (19.3%) are a minor crop, and aerial applications (33.2%) now require a derogation within the EU.

A literature search was carried out for mass‐based quantification of spray drift losses to surface water in catchments to check the plausibility of the derived release factor for surface water.

The coformulant maximum annual application rate (i.e., expressed in kg/ha) can be considered an operational condition in REACH terminology (ECHA 2012). Risk management measures (RMMs) such as spray drift reduction (e.g., buffer zones, drift‐reducing coformulants) are usually formulation specific and driven by the relevant active ingredient hazard profile and crop. As a result for the development of a SpERC, no RMM can be assumed for the generic coformulant case. Such RMMs could be taken into account in substance‐specific refinements, but this would require specific information on individual formulations, the crops applied to, and the tonnages used therein. In practice this detailed information would be available only for downstream user chemical safety assessments or in specific product risk assessments conducted under PPP regulations.

## RESULTS

**Figure 1:**
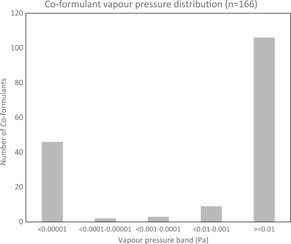
Vapor pressure distribution of a 166‐coformulant subset of the coformulants currently on the European market. Pa = Pascal.

The distribution of the vapor pressures of the investigated coformulants is shown in Figure 1, with a clear U‐shaped distribution. Based on this subset of the coformulants currently on the European market, for the purposes of environmental risk assessment, most substances can be expected to be categorized as either “volatile” or “nonvolatile,” and only a minority fall within the transition between these 2 categories.

### Spray application

#### Release to air

The release factors to air, F_air_, proposed in USES 4.0, were adopted as the starting point for developing vapor pressure dependent subSpERCs because they had preexisting regulatory acceptance (Supplemental Data Table S1a). The vapor pressure range encompassed by the USES 4.0 variable release factors is that expected to span the transition from nonvolatile to volatile behavior of substances in the environment (Cousins et al. 1999). Conceptually the release factor to air is intended to encompass the initial dynamic processes during spraying (high surface area mist) and shortly after deposition that lead to partitioning to the atmospheric compartment. Taking the very conservative precedent from USES, this was taken as within 24 h.

**Figure 2:**
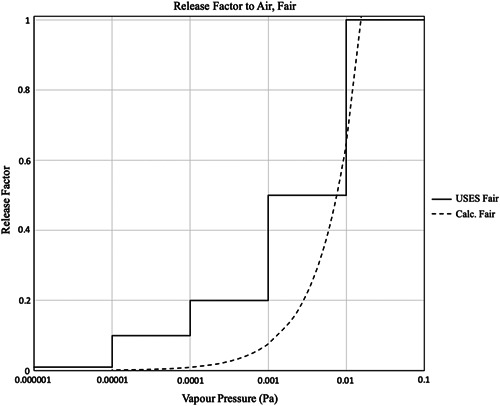
Comparison between the USES 4.0 release factors to air from pesticide use, and calculated using F_air(Calc)_ = 24 × ER/100 000 × AR, where ER is given by Equation 1, and a single application rate 1 kg/ha. Release factors above the dashed line are conservative in comparison to the predicted model evaporation rate. AR = application rate; ER = evaporation rate; Pa = Pascal; USES = Uniform System for the Evaluation of Substances.

Using Equation 1, the calculated vapor pressure–dependent volatilization at 24 h at 1 kg/ha single application rate was calculated and plotted in Figure 2. The USES 4.0 F_air_ values thus appear to be a reasonable, conservative approximation of a continuous function transitioning from the nonvolatile to volatile regime, and have been adopted unmodified into the subSpERC in Table 1. The release factor band 0.001 to 0.01 Pa appears to be underestimated in comparison to the calculation, for a very narrow range, just below 0.01 Pa. The apparent 20% underestimate is not considered to be significant when the 24‐h emission duration is considered, in comparison to the much shorter actual field activity. Similarly, potential factors arising from variable conditions such as temperature and mixture effects should be sufficiently covered by this conservatism, as well as the experimental data sets used by van Wesenbeeck et al. (2008) to derive Equation 1.

#### Release to soil

The most important exposure determinant affecting spray emission to the agricultural soil compartment is crop interception. This is the fraction of the applied substance that is retained on the crop, and as a result does not reach the soil surface. Because the values are crop specific, any average would need to make assumptions on regional crop distributions and timing of application. Alternatively the simplest approach is to take the worst case where there is no interception by the crop, that is, all of the sprayed formulation reaches the soil. This may occur when a formulation is used prior to crop emergence, but it is clearly not the most frequent scenario.

The coformulant dose that reaches the soil can also be significantly reduced due to volatilization from spray droplets, spray drift, and plant surfaces and soil within the first 24 h (Guth et al. 2004; van Wesenbeeck et al. 2008). Nevertheless, despite some expected dependency on F_air_ and F_surface water_, these factors have been omitted in the more conservative Version 4 of the SpERC, with the worst case of complete emission to soil assumed to be up to a cutoff vapor pressure of 0.01 Pa. Above 0.01 Pa, full volatilization of the substance is assumed within 24 h, effectively the inverse of Figure 2, and the value for F_soil_ is set to 0 (Table 1).

#### Release to surface water

Only spray drift is considered to be direct emission to surface water, and thus relevant to the release factor.

The surface area of the adjacent water body in the FOCUS scenario constitutes 1% of the area of the treated field (10 000 m^2^ for the field, and 100 m^2^ for the water body; FOCUS 2003). Therefore, even if the water body were oversprayed at the same rate as the field, only 1% of the applied substance mass would enter the water body. Assuming that direct overspray does not in fact occur (not good agricultural practice), and using the reasonable worst‐case spray drift for orchards of 15.7%, the fraction of the applied substance entering the water body reduces to F_surface water_ = 0.00157, or rounded to 0.002 (Table 1). Such a calculation does not take into account the proportion of agricultural land that is adjacent to waterbodies and available for spray drift and is thus effectively calculating the amount of substance released to surface water if all agricultural land in the EU consisted only of orchards located within 100 m of a water body.

Of the relevant articles identified in the literature search investigating loss of PPPs at the regional catchment or landscape scale (Dabrowski and Schulz 2003; Gevaert et al. 2008; Holvoet et al. 2008; Dabrowski and Balderacchi 2013; Lefrancq et al. 2013), two contained quantitative data (Huber et al. 2000; Bach et al. 2005). The general conclusions that can be drawn from these papers are that for field crops at the regional scale, spray drift is a minor exposure route for surface water compared to runoff, and that in orchards and vineyards, spray drift could be of increased importance as an exposure route. It should be noted that in the context of the EU TGD model, the regional scale is defined as an area of 200 × 200 km^2^ and is not necessarily congruent with the spatial scales expressed in these studies. Huber et al. (2000) conducted geographic information system (GIS) calculations for the use of 42 active ingredients across Germany, based on product usage data from 1994. They made quantified predictions as to the loss to surface water of substances via runoff, drainage, and spray drift at the national scale. Their findings included a predicted total annual loss of 13 900 kg active substance with 9060 kg via runoff (65.2% of total loss), 1490 kg via drainage (10.7% of total loss), and 3350 kg via spray drift (24.1% of total loss). Spray drift varies by application method, with significantly higher loss rates encountered for air blast methods frequently used in orchards and vineyards compared to field crops.

For field crops, spray drift accounted for only 90 kg of losses, which was <1% of the total predicted losses. However, for orchard and vineyards, spray drift was approximately 80% of the total losses. The paper by Huber et al. (2000) suggests that for field crops only runoff and drainage need to be considered and that spray drift (at the regional scale) makes a negligible contribution to emission to surface water. However, this may not be the case for areas that have a high concentration of orchards and vineyards, where loss via spray drift could be more significant, thus supporting the worst‐case spray‐drift rate selected to derive the surface water release factor. Huber et al. (2000) did not report the applied dose used in their analysis, and losses as a fraction of applied dose (i.e., release factor) cannot be calculated for direct comparison. However, cumulative losses for runoff for individual active substances were reported to be in the range 0.01% to 0.42% of the application rate.

Bach et al. (2005) conducted GIS calculations for the use of 59 active substances across Germany, based on product sales data from 2000, but only for field crops. They made quantified predictions as to the loss to surface water of substances via runoff, drainage, and spray drift at the national scale. Their findings confirm those of Huber et al. (2000) with respect to the relative unimportance of direct emission by spray drift for field crops at the regional scale. They also help to quantify how much might be lost via these processes as a percent of applied dose. Only 0.11% of the applied dose was predicted to be lost from field crops via runoff (within the range reported by Huber et al. [2000]), followed by 0.0013% via drainage, and 0.0003% via spray drift, demonstrating the SpERC release factor for surface water is conservative for field crops.

#### Release to waste

Product labeling provides guidance regarding appropriate disposal of PPPs. It is recommended that used containers are triple or pressure rinsed, or rinsed with a system that is an integral part of the sprayer, prior to package disposal. Washing in this manner has been demonstrated to retain <0.01% of the formulation in the container (Lavers 1993; Mostade et al. 1997). The wash water is added to the sprayer at time of filling and thus is already accounted for within the overall release factors. Properly rinsed containers may be disposed of as nonhazardous plastic packaging. Furthermore, collection and recovery schemes for crop protection containers exist in many European countries.

### Granular or solid application

#### Release to air

For granular products and treated seeds, it was assumed that use of highly volatile coformulants would be unlikely: These are typically liquids that cannot be found in significant concentrations in a solid product, and low melting point solids or solids prone to sublimation would pose product stability issues. The special case of volatile solvents potentially used in seed treatment formulations has also been recognized and included within the scope of the SpERC for spray applications. This is because the solvent is lost to air on drying of the seed coating. Potential volatilization during application of granules and treated seeds was assumed to be negligible, unlike a high surface area spray mist with potential aqueous coevaporation (FOCUS 2008; Delmaar and Bremmer 2009). The potential for soil incorporation of granules, and as a requirement for treated seeds, would also hinder volatilization processes. Creation of a detailed vapor pressure–dependent subSpERC was thus considered unwarranted. The release factor F_air_ was set to 0 (Table 1), also for substances with vapor pressure ≥0.01 Pa.

#### Release to soil

For granular products and treated seeds, the worst case is no interception by the crop (i.e., direct application to the soil), but unlike spraying this would be considered the normal scenario. As a result, full emission to the soil compartment was assumed, including for substances with vapor pressure ≥0.01 Pa, should they be used (F_soil_ = 1) (Table 1).

#### Release to surface water

Direct emission to surface water for granules and treated seeds was set to 0 (F_surface water_ = 0), following the FOCUS guidelines (FOCUS 1997). Although dust generation during broadcast applications has been posited as being potentially relevant for active substance assessments (EFSA 2004), there is currently no agreed data (drift tables) or risk assessment approach for this scenario in PPP assessments. Furthermore, such approaches have focused on determining localized peak concentrations for risk assessment, which is of limited relevance to the fraction of the regional tonnage emitted to surface water needed for the SpERC release factor. An additional very small nonzero release factor representing a hypothetical low tonnage direct emission would be unlikely to meaningfully change the PEC_regional_.

#### Release to waste

Product labeling provides guidance regarding appropriate disposal of PPPs and emptied containers. Specific estimates of residues remaining in packaging for solid formulations (granules or treated seeds) are not available. The emission scenario document for plastic additives suggests that for powders of particle size >40 µm, 0.01% could be expected to remain in packaging (OECD 2009). Based on an analogous scenario (i.e., solid substances present as granules in a plastic container), the release factor was considered suitable for “read‐across” (Tolls et al. 2015).

## DISCUSSION

The latest ECPA SpERCs for application of granules and spray application of PPP formulations were designated 8d.1.v4 and 8d.2.v4, respectively, following the standardized naming nomenclature, derived from ERC 8d (ECHA 2015). Both are currently in Version 4, with the evolution of previous SpERC versions presented in Supplemental Data Tables S1b and S1c.

The first versions of the ECPA SpERC set the release factor to surface water to 0, on the basis that PPP approvals under EU Regulation (EC) Nr 1107/2009 include specific labeling instructions designed to prevent emission to waste and surface water, and intentional release would be noncompliant with that legislation (Supplemental Data Table S1b) (EC 2009). The SpERC was revised in Version 3 and assumed that in the worst case unintentional spray drift to surface water occurs (Supplemental Data Table S1c). This also resulted in a conceptual alignment with the assessment of local environmental exposure in the ECPA LET model, where spray drift is assumed to occur. Because the concentration in air at the regional scale is not a standard target for risk characterization (PNEC_air_ is usually not available) and only indirectly influences the PEC_regional_ in the other compartments via redeposition, mass balance and conservatism were maintained by reducing F_air_ rather than F_soil_. Despite the conceptual improvement, because of the conservative approach used to model runoff in the EU TGD, only very small changes to the calculated PEC_regional_ have been seen in practice, compared to the preceding versions that assume no direct emission to surface water.

The most recent update presented here (Table 1) recognizes that although maintaining mass balance between compartment release factors makes scientific sense (i.e., the tonnage emitted cannot be greater than the amount used in a PPP), excessive conservatism in 1 value may lead to underprediction in another compartment. For this reason the vapor pressure–dependent values for F_air_ were decoupled from F_soil_ and F_surface water_, and reverted to the original USES 4.0 values. Figure 1 illustrates that the evolution of the vapor pressure–dependent values for the ECPA SpERCs are unlikely to impact risk assessments of many registered substances because most coformulant vapor pressures are either higher or lower, and empirical experience is that PEC_regional_ is relatively insensitive.

### Local scale assessment

The assessment of PEC_local_ for coformulants lies outside the applicability domain of the current EU TGD–based models (direct emission to agricultural soil is not considered for widespread uses) and therefore needs to be carried out with a suitable alternative, for example, the ECPA LET model. Depending on the EU TGD implementation, the local release factor should be manually set to 0 (e.g., EUSES), or the calculated PEC_local_ simply discarded if this is not possible. The latest version of Chesar (version 3) automatically disables the calculation of PEC_local_ when “agricultural soil” is set (ECHA 2019a).

### Additional SpERC differentiation

On a tonnage basis, the vast majority of PPPs are applied to agricultural fields outdoors, which results in direct emissions of coformulants into the environment. In comparison, only a minor tonnage is applied “indoors,” for example, in greenhouses. The ECPA SpERCs are based on the realistic worst‐case emissions expected for the outdoor scenarios. Any indoor emissions are ultimately expected to also reach the relevant modeled environmental compartments, but with a temporal delay (e.g., air exchange) or with an equivalent (e.g., soil) or smaller release factor (e.g., no spray drift to surface water). Where there is uncertainty associated with the type of indoor application, the guidance on risk assessment for PPPs in the EU generally assumes that the risk assessment for field uses represents the worst case (EFSA 2014). The characterization of environmental emissions resulting from the outdoor use of PPP with the ECPA SpERCs is therefore regarded as inherently covering indoor uses (ECHA 2016a). In terms of modeling a PEC_regional_, the driver for differentiating a SpERC is that the contributing environmental scenario for an identified use is inherently different from another use scenario, resulting in different environmental releases. In the case of the ECPA SpERCs, given that indoor uses are unlikely to result in higher environmental exposure compared to field applications at the regional scale, further differentiation with 2 additional indoor‐specific SpERCs, based on ERC 8a, is not considered justified.

The 2 ECPA SpERCs do not currently differentiate professional and consumer uses of PPPs. In terms of tonnage, it was anticipated that the consumer and/or amenity use made up only a small portion of the total use of PPPs. An analysis of the usage of pesticides in the United Kingdom (UK) in the year 2012, for example, showed that amenity use accounted for less than 4% of the total pesticide use in the UK (Goulds 2015). Therefore, it is reasonable to assume that the realistic worst‐case release factors defined for professional use would not be higher for amenity and/or consumer use, and that the small tonnages involved would not lead to significantly different calculated PECs.

### Risk management measures

The ECPA SpERCs do not assume the use of any specific RMMs. Risk management measures such as buffer strips, although possible to specify as a justification to further reduce spray drift (and hence the surface‐water release factor), would inevitably conflict with the use instructions of existing authorized products, which did not specify these for certain formulations or crops. Considering the extremely conservative assumptions made in deriving the release factors to surface water, such additional RMMs would not be meaningful. Product labeling is assumed to be followed for waste disposal, and any intentional PPP release to surface water or a sewage treatment plant is clear product misuse.

### Further refinements to release factors

In general the ECPA SpERCs have traded uncertainty for conservatism by selecting the worst‐case values. This offers the possibility of further refinement for substance‐specific risk assessments, provided appropriate justification can be given.

On a case‐by‐case basis, F_air_ could be refined on the basis of vapor pressure, particularly for substances that fall in the transition from nonvolatile to volatile. Similarly, F_soil_ could benefit from further information on crop interception. In both cases it would, however, be expected that the need for refinements to the risk assessment would be driven by the LET and PEC_local_ rather than by PEC_regional_.

For the ECPA SpERCs, further refinements could be envisaged to justify a smaller release factor to surface water on the basis of land‐use patterns through spatial modeling, for example, taking into account crop type, associated drift rates, and proximity to surface‐water bodies. Different release factors for regional and local scales could be conceived reflecting regional‐scale crop diversity and local‐scale homogeneity. However, empirical testing of the sensitivity of the EU TGD model to F_surface water_ at the regional scale suggested little benefit was to be gained from further refining surface‐water release factors, in terms of obtaining less conservative estimates for PEC_regional_. It must be emphasized that the values for F_surface water_ presented here (Table 1) are a fit‐for‐purpose, EU TGD–specific overestimation that could require further refinement in future for use with more sensitive regional‐scale or local‐scale models.

## CONCLUSION

The ECPA has developed 2 sector‐specific SpERCs to aid in the regional‐scale risk assessment of substances used as coformulants in PPPs. One SpERC covers formulations that are applied in granular form or treated seeds. The second covers formulations that are applied as sprays and takes account of the vapor pressure of the substance. Both SpERCs provide release factors that reflect the typical application methods of PPPs and the substance properties that are used in these formulations. Calculations have supported the spray application subSpERC transition from nonvolatile to volatile behavior, based on vapor pressure. Use of the ECPA SpERCs allows the contributions from coformulant emissions from PPPs to be fully accounted for in the REACH regional‐scale risk assessment of a given substance, and the regional background from other nonPPP uses to be accounted for in a local‐scale assessment of the use as a coformulant. The use of SpERCs limited to the calculation of regional‐scale PECs with EU TGD–based models, in combination with a customized local‐scale exposure model, is a potentially useful approach for other sectors looking to address scenarios that lie outside the scope of the standard EU TGD.

## Disclaimer

The ECPA SpERCs were developed by an expert group formed from the member companies of the European Crop Protection Association and tasked with developing a methodology for assessing coformulants under REACH. Cambridge Environmental Assessments received direct financial support for the development of the LET model. All other authors participated in the expert group during the normal course of their employment. The ECPA member companies are commercial entities which produce and market products that are subject to regulation by REACH. The authors have responsibility for the writing and contents of the manuscript, and the views expressed in this article are those of the authors and do not necessarily represent the views or policies of the ECPA, or their respective employers.

## SUPPLEMENTAL DATA

**Table S1a.** USES 4.0 pesticide module release factors to air

**Table S1b.** ECPA SpERC Version 2

**Table S1c.** ECPA SpERC Version 3.

## Supporting information

This article contains online‐only Supplemental Data.

Supporting information

## Data Availability

The ECPA‐developed LET model, SpERCs, and documentation are available at https://www.ecpa.eu/pre‐market‐resources‐for‐industry/reach‐registration‐evaluation‐authorisation‐and‐restriction‐chemicals.
