# Supplementary material for: REACH Specific Environmental Release Categories for Plant Protection Product Applications
Source: Integr Environ Assess Manag. 2020 Mar 25;16(4):472–80. doi: 10.1002/ieam.4251 (PMC7317189; doi:10.1002/ieam.4251)
Supplement: Supplementary file 1 — Supporting information [file IEAM-16-472-s001.docx]

**Table S1a.** USES 4.0 pesticide module release factors to air.

RIVM 2002, Table A-2, p211 and Table D-3, p318:

| **Vapour pressure band (Pa)** | **F_air_ (-)** |
| --- | --- |
|  |  |
| >0.01 | 1 |
| 0.001-0.01 | 0.5 |
| 0.0001-0.001 | 0.2 |
| 0.00001-0.0001 | 0.1 |
| <0.00001 | 0.01 |

**Table S1b.** ECPA SpERC Version 2.

| **Vapour pressure band (Pa)** | **F_air_ (-)** | **F_soil_ (-)** | **F_surface water_ (-)** |
| --- | --- | --- | --- |
| Release factors spray applications (SpERC 8d.2.2). | | | |
| >0.01 | 1 | 0 | 0 |
| >0.001 - 0.01 | 0.5 | 0.5 | 0 |
| >0.0001 - 0.001 | 0.2 | 0.8 | 0 |
| >0.00001 - 0.0001 | 0.1 | 0.9 | 0 |
| < 0.00001 | 0.01 | 0.99 | 0 |
| Release factors for application as granules or treated seeds (SpERC 8d.1.2). | | | |
|  | 0 | 1 | 0 |

**Table S1c.** ECPA SpERC Version 3.

| **Vapour pressure band (Pa)** | **F_air_ (-)** | **F_soil_ (-)** | **F_surface water_ (-)** |
| --- | --- | --- | --- |
| Release factors spray applications (SpERC 8d.2.3). | | | |
| >0.01 | 0.998 | 0 | 0.002 |
| >0.001 - 0.01 | 0.498 | 0.5 | 0.002 |
| >0.0001 - 0.001 | 0.198 | 0.8 | 0.002 |
| >0.00001 - 0.0001 | 0.098 | 0.9 | 0.002 |
| < 0.00001 | 0.008 | 0.99 | 0.002 |
| Release factors for application as granules or treated seeds (SpERC 8d.1.3). | | | |
|  | 0 | 1 | 0 |
